# The role of loneliness in university students’ pathological Internet use – a web survey study on the moderating effect of social web application use

**DOI:** 10.1007/s12144-021-02455-3

**Published:** 2021-11-12

**Authors:** Andreas Reissmann, Klaus W. Lange

**Affiliations:** Institute of Psychology, University of Regensburg, 93040 Regensburg, Germany

**Keywords:** Loneliness, Moderated mediation, Pathological Internet use, Social web application use

## Abstract

The present study investigated the role of social web application use in the association between loneliness and pathological Internet use. A sample of university students (n = 445) completed an online survey of their loneliness and Internet use, including an assessment of pathological Internet use level. Based on existing theory and empirical findings, loneliness was expected to be indirectly associated with pathological Internet use through social-compensatory Internet use motives. The strength of this indirect effect was hypothesized to be moderated by levels of social web application use. Results pointed to the specificity of social-compensatory use motives in mediating loneliness effects on pathological Internet use, while the size of these effects was moderated by quantity of social web application use. Findings suggest that lonely people with higher levels of social web activity show a stronger social-compensatory use orientation, which translates to higher levels of pathological Internet use. Implications and limitations of these findings are discussed and several suggestions for future studies are made.

## Introduction

Loneliness can be defined as the disquieting experience of a perceived internal distance between oneself and others and is conceptually linked to the desire for and eventual attempts to reconnect with others (Schwab, 1997). Loneliness and underlying social contact problems are highly prevalent in emerging adult as well as university student samples (Qualter et al., 2015). Excessive and maladaptive uses of the Internet have been linked to social isolation from early in the Internet era (Kraut et al., 1998), with loneliness regarded as both an antecedent and a result of such excessive use of the Internet (Moretta & Buodo, 2020).

When associated with a loss of control of the use behavior and resultant problems regarding time management as well as with social problems and craving for the use of the Internet, such excessive use has been discussed as part of a syndrome of pathological Internet use (Kraut et al., 1998; Tran et al., 2017b). A large set of sociodemographic (e.g. male gender, single status, student status), psychosocial (e.g. impulsivity, introversion, low self-esteem, loneliness, homesickness) and psychopathological (e.g. depressive symptoms, social anxiety disorder, attention-deficit/hyperactivity disorder (ADHD), aggression, substance use) correlates have been associated with pathological Internet use (Ho et al., 2014; Kuss et al., 2014; Sun et al., 2012). Moreover, pathological Internet use has been associated with reductions in physical activity (Dang et al., 2018), poor sleep quality (Zhang et al., 2017) and reduced levels of health-related quality of life (Tran et al., 2017a). The syndrome has been shown to be more prevalent in adolescent and emerging adult samples, including students (Durkee et al., 2012; Kuss et al., 2014; Zhang et al., 2018), with an estimated prevalence rate of approximately 4.4% in European adolescents (Durkee et al., 2012). Importantly, however, there is no universally accepted definition of the syndrome and different diagnostic criteria have been used to establish a syndromic diagnosis (Musetti et al., 2016), certainly contributing to highly divergent prevalence estimates (for Europe: 1 to 21%, see Duong et al., 2020) and large numbers of identified correlates in the literature (Duong et al., 2020; Kuss et al., 2014).

These inconsistencies are also reflected in an ongoing debate in the scientific community and a general lack of consensus regarding the validity of diagnostic categories pertaining to pathological forms of technology use, such as (Internet) gaming disorder (Aarseth et al., 2017; Ferguson & Colwell, 2020). While ‘Internet gaming disorder’ has been formally included in the research appendix of the fifth edition of the Diagnostic and Statistical Manual of Mental Disorders (DSM-5, American Psychiatric Association, 2013), the broader term ‘gaming disorder’ has been formally included in the eleventh edition of the International Classification of Diseases (ICD-11, World Health Organization, 2020). This formal inclusion should not, however, obscure the fact that the available evidence pertaining to important questions such as the temporal stability of the syndrome (Rothmund et al., 2016), the separability from (underlying) psychopathological problems (Aarseth et al., 2017) or even the establishment of sound diagnostic (screening) procedures across age and cultural groups is far from conclusive (Petry et al., 2020). While this holds true for (Internet) gaming disorder as a more specific kind of excessive interactive media use, this holds even more true for the more general umbrella term of pathological Internet use (Petry et al., 2020), which makes difficult the scientifically sound establishment of pathological Internet use as a psychiatric disorder (Duong et al., 2020; Musetti et al., 2016). Therefore, pathological Internet use has not been included as a diagnosis neither in the DSM-5 nor the ICD-11.

### The role of loneliness in pathological Internet use

Etiological accounts of pathological Internet use try to account for the many psychosocial and psychopathological factors associated with the syndrome (see Kuss et al., 2014 for review) in order to arrive at a better understanding of the pathways involved in the development of the disorder. As an example, the cognitive-behavioral model by Davis (2001) and Caplan (2002, 2003) proposes that psychosocial problems such as loneliness or social anxiety set people at higher risk for developing pathological forms of Internet use.

These predictive relationships between loneliness and pathological Internet use have been established on an empirical basis in both cross-sectional (Brand et al., 2014; Morahan-Martin & Schumacher, 2003) and longitudinal studies (Jia et al., 2018). There are also indications of bidirectional effects, however, as reviewed by Moretta and Buodo (2020). Within the scope of the cognitive behavioral model of pathological Internet use, the (social) provisions of the Internet environment are hypothesized to be especially appealing to and entail the development of maladaptive cognitive processes and expectations (e.g. “*Without the Internet, I am no one!*”) in psychosocially vulnerable persons. By repetitively engaging in online social activities and experiencing the gratification of social needs unmet in in-person life, the Internet might become an important means of satisfying social needs, thereby setting in motion a vicious cycle of problematic use behavior. Lonely and socially isolated individuals may experience the social aspects of the Internet as a central means for the fulfillment of their social needs. This may put them at risk for the development of pathological Internet use through increasing use for social-compensatory purposes (Davis, 2001). Social-compensatory use refers to Internet use that aims at the fulfillment of social needs unmet in the offline world. However, there are also other Internet use motives other than social ones such as fun and entertainment or information access (Ruggiero, 2000). Some of these use motives and expectations regarding Internet use (e.g. entertainment/relaxation, arousal/emotion regulation and social compensation) have been shown to be associated with pathological Internet use (Brand et al., 2014; Kim & Haridakis, 2009; Morahan-Martin, 1999). Contrary to this, Internet use driven by information and education purposes has been shown to be negatively associated with pathological Internet use (Bozoglan et al., 2014). Therefore, investigating the specificity of etiologic pathways towards pathological Internet use in lonely persons represents an important research topic within the scope of the cognitive behavioral model.

Loneliness itself has been shown to be associated with emotion-regulatory use motives (Brand et al., 2014; Caplan, 2002, 2003; Morahan-Martin & Schumacher, 2003) as well as social-compensatory use motives (Brand et al., 2014; Caplan, 2002, 2003; Morahan-Martin & Schumacher, 2003; Reissmann et al., 2018), while there is conflicting evidence regarding pastime user orientation (Morahan-Martin & Schumacher, 2003, but not Hollenbaugh & Ferris, 2014). Therefore, given the multiple associations with a diverse range of use motives, it seems that empirical investigations of the links between loneliness and Internet use motives should take a broad form and include multiple motives into their research design. This would also allow for an analysis of the specificity of loneliness effects on social compensatory use motives, as predicted within the scope of the cognitive behavioral model of pathological Internet use (Caplan, 2002, 2003; Davis, 2001). Moreover, studying the contingencies upon actual Internet use behavior would seem to be important as well in this regard: Only by using the Internet in certain ways (e.g. by using social web applications), certain types of gratification could be obtained (e.g. satisfaction of intimacy needs). One might hypothesize that for lonely persons who repetitively engage in social web applications, the Internet may become an especially important way of relating with others. This has also been shown within everyday contexts, where state feelings of loneliness were more predictive of subsequent use of the social media service Facebook in trait-lonely persons (Reissmann et al., 2018). To the knowledge of the authors, however, no empirical investigation within the scope of the cognitive behavioral model has as-yet investigated the possibility of multiple motivational mediators of loneliness effects on pathological Internet use levels. Moreover, no such study has tried to integrate the study of moderating effects pertaining to the actual use of social web applications in explaining the links between loneliness and social-compensatory Internet use motives. Therefore, the following research questions and hypotheses are posited:

## Research questions and hypotheses

Research question 1 of this study is concerned with the role loneliness plays in Internet use motives.

- **Hypothesis 1a:** Loneliness will also be related to Internet use motives other than social-compensatory ones.
- **Hypothesis 1b:** The strength of association between loneliness and social-compensatory Internet use motives will be specifically contingent on the actual amount of social web application use a person is engaging in.

Research question 2 concerns the underlying (i.e. mediating) mechanisms linking loneliness and pathological Internet use.

- **Hypothesis 2a:**Different use motives will (*i.*) be associated with pathological Internet use and (*ii.*) act as mediators of loneliness effects.
- **Hypothesis 2b:** The size of indirect loneliness effects, as mediated by social-compensatory Internet use motives, will be contingent on the level of social web application use employed.

## Methods

The study was approved by the local ethics committee at the University of Regensburg (Study Code: 15-101-0107) and complied to the code of ethics of the World Medical Association (Declaration of Helsinki). Data collection for this study was conducted and completed prior to the coronavirus disease 2019 (COVID-19) pandemic.

### Sample

A sample of university students at universities in German-speaking countries was recruited via local student representatives, who were asked to disseminate the web link to the online survey and an appeal to solicit contribution from students to the study. A total of 445 participants completed the survey and fulfilled the data requirements for the subsequent analyses (minimum age of 18 years, enrollment as a student at a university, providing full information, German native speaker). Mean age of the sample was approximately 24 years (M = 23.84; SD = 3.70), additional sociodemographic information of the final sample can be found in Table 1.

**Table 1:** Sociodemographic information of the study sample

		N (%)	
Gender	female	270	(60.7%)
	male	175	(39.3%)
Marital status	married	15	(3.3%)
	unmarried/divorced	430	(96.6%)
Partner status	in relationship	249	(56%)
	single	196	(44%)
Household arrangement	alone	114	(25.6%)
	at parents’	71	(16%)
	with spouse	94	(21.2%)
	shared flat	159	(35.7%)
	other	7	(1.5%)
Residential area	metropolitan (above 100,000 inhabitants)	238	(53.5%)
	urban (20,000 to 100,000 inhabitants)	138	(31%)
	small town (2,000 to 20,000 inhabitants)	42	(9.4%)
	rural (less than 2,000 inhabitants)	27	(6.1%)
Field of study	linguistic and cultural studies	62	(13.9%)
	social sciences	83	(18.7%)
	engineering	53	(11.9%)
	theatre and arts	7	(1.6%)
	teacher training program	50	(11.2%)
	medicine and health care	35	(7.9%)
	natural sciences	136	(30.6%)
	law and business sciences	14	(3.1%)
	other fields of study	5	(1.1%)
Highest level of education obtained	university-entrance diploma	280	(62.9%)
	bachelor’s degree	132	(29.7%)
	master’s degree	29	(6.5%)
	other	4	(0.9%)
Occupational status	no gainful occupation	201	(45.1%)
	mini/ irregular jobs	179	(40.2%)
	paid form of university studies	17	(3.8%)
	part-/full-time employment	48	(10.8%)

### Instruments

In the following, a brief description of the instruments used and the implementation of the online survey will be given.

#### Implementation of Limesurvey™

The online survey was implemented using the freely available online-survey application Limesurvey™ (LimeSurvey Project Team & Schmitz, 2012) in Version 1.92+ on a webserver of the University of Regensburg. Care was taken to ensure the confidentiality of the recorded data and anonymity of participants. In order to enhance the rate of completed surveys, participants were allowed to store their responses temporarily for later completion.

#### Internet usage and preferences

Immediately after collecting sociodemographic data, information with respect to participants’ Internet use was collected (inter alia the types of Internet access used, usage frequency and duration of different Internet activities). Participants were solely surveyed regarding their off-work Internet use for private purposes. Since participants were allowed to choose from a predefined list of 12 different Internet activities as well as to add additional activities themselves, a recoding scheme was implemented to form seven main categories of online activities (see Table 2). For the usage duration parameters, the durations (estimated in hours of usage per week by the participant) were summed for all the sub-categories allocated to a specific main category (see Table 2).

**Table 2:** Overview of the seven main categories of Internet use and their allocated sub-categories

Main category	Allocated sub-category	Hours used per week M (SD)
SOC	**keeping in touch with acquaintances**	9.49 (11.75)
	**establishing new acquaintances**	
	**active participation in bulletin boards**	
	*specific forms of communication*	
AV	**watching/downloading videos**	11.51 (12.24)
	**listening to/downloading music**	
	*specific forms of audiovisual entertainment*	
GAME	**playing online games**	2.58 (6.03)
PORN	**watching pornographic contents**	.71 (1.86)
INF	**any form of directed information search**	5.41 (5.66)
	*news reading and concrete information purposes*	
SURF	**any form of undirected Internet use/ information search**	4.21 (6.31)
LP	**online banking**	.96 (1.78)
	**online shopping**	
	*private organizational and productive activities*	
	*IT- and web-related activities*	
	*digital authoring*	
Overall use	collapsed across categories	34.88 (28.87)

*Bold items in the “allocated sub-category” column represent the predefined categories that participants could select. Note that only eleven subcategories are listed within this table; M: mean; SD: standard deviation; SOC: social web application use; AV: audiovisual entertainment service use; INF: specific information and learning service use; LP: productive and everyday life-practical services; SURF: web surfing and undirected information search; GAME: online game use; PORN: pornographic content consumption.*

#### Motives for Internet use

In order to assess different motivational aspects regarding Internet use, a set of 16 items, each comprising a specific reason for the use of the Internet was created. Nine items were taken from an established instrument (Engel & Best, 2010) used within the longitudinal study “mass communication”, which is carried out on behalf of the two major public television broadcasters in Germany. Each of the 9 item comprises a specific reason for the use of the Internet (e.g. “*because it is fun to me*”; “*because I want to get informed*”; “*because I want to distract myself*”) and respondents are asked to rate the degree each statement fits their Internet use. This item pool was complemented with seven ad hoc items reflecting additional motivational aspects of Internet use (e.g. “*because this is where I can be the real me*”), as listed in Table 4.

The initial pool of 16 items was analyzed using exploratory factor analysis (EFA) performed with the “psych” package (version 2.0.8) in RStudio (version 1.4.1106; R version 4.0.5). This exploratory analysis was complemented with a confirmatory factor analysis (CFA) in order to validate the established factorial structure. The sample was randomly split into two groups (n_EFA_ = 219; n_CFA_ = 226). The EFA was conducted using half of the study sample (n_EFA_ = 219) with principal axis factoring as extraction method. The correlation matrix was based on polychoric correlations (estimated by the two-step procedure), and Oblimin rotation was employed to allow for correlated factors. Based on the guidelines specified by Costello and Osborne (2005), items had to fulfill the following criteria in order to be retained in the final model: item communalities had to be 0.40 or higher, items had to load at least at 0.32 on one factor and show no cross-loadings greater than 0.32 on any other factor. Furthermore, the number of factors to be retained was based on the results of Horn’s Parallel Analysis (Horn, 1965) and Velicer’s MAP criteria (Velicer, 1976), as suggested by Costello and Osborne (2005). Although Parallel Analysis and MAP results differed initially (with MAP criteria suggesting a three factor solution, while Parallel Analysis pointed towards a four factor solution), the methods clearly pointed towards a three factor solution after excluding items with low communalities. Thus, the three factorial solution was further investigated. Applying the above mentioned criteria led to the exclusion of seven items (see Table 4). The final solution comprised three factors (represented with three items each), which explained a total of 59.4% of the variance among items (see Table 4). The first factor was named “*fun & relaxation*” (M1_fun) (e.g. “*because it is fun to me”)* as it contained items relating to fun, entertainment, and relaxation uses of the Internet. The second factor was named “*social & personal unfolding*” (M2_soc) (e.g. “*because then I do not feel lonely*”) as it contained items relating Internet use to the alleviation of loneliness and the realization of personal identity and social needs. The third factor was named “*information & learning*” (M3_inf) (“*because I want to get informed*”) since it contained items relating to Internet use for informational purposes and personal development through learning.

**Fig. 1:**
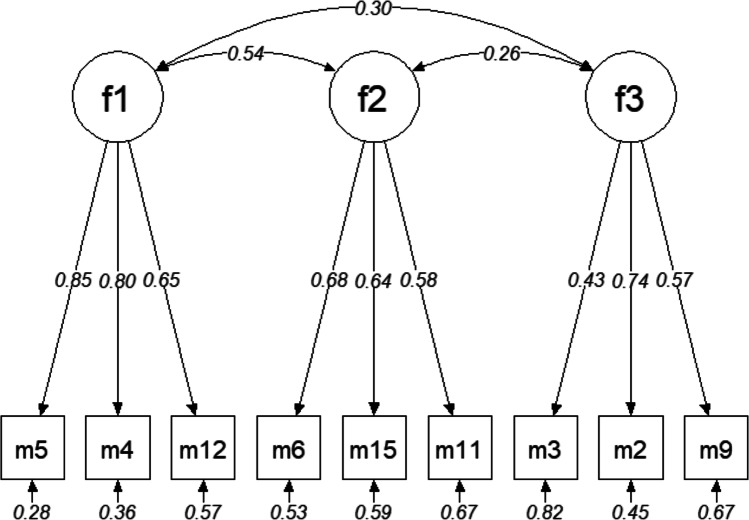
CFA model with correlated factors and standardized factor loadings

Subsequently, a CFA was conducted aiming at the replication of the three-factorial structure in the final 9 item set of the EFA, using the second half of the sample (n_CFA_ = 226). Therefore, a CFA model was specified using the “lavaan” package (version 0.6-9) that specified three item indicators per factor, based on the highest factor loadings in the EFA (see Table 4). Moreover, correlations between factors were included in the model. This model was estimated using diagonally weighted least squares (DWLS) as estimation method due to the ordinal scale of the item indicators and the violation of the assumption of multivariate normality (Li, 2016). The CFA confirmed the postulated three-factorial structure, with factor loadings ranging between .43 – .85 and inter-factor correlations ranging between .26 –.54 (see Fig. 1). The absolute and incremental measures of Model Fit pointed towards adequate model fit (CFI = .979; SRMR = .061; RMSEA = .046, 90%-CI: .000 – .076, p = .551). This was confirmed by the Model Chi Square, which showed an insignificant result (χ2 = 35.379, df = 24, p = .063).

Based on these results, factor-based scores were calculated by summing raw item scores for each of the three subscales (DiStefano et al., 2009). Higher scores indicated that a subject’s Internet use was more strongly characterized by the respective motivational dimension.

#### Short Version of the Internet Addiction Test (sIAT)

In order to assess the presence and severity of symptoms related to pathological Internet use, a shortened form of the Internet Addiction test encompassing 12 items was administered (Pawlikowski et al., 2013). This scale is an updated and streamlined version of the scale measure originally introduced by Young (1998) and has been shown to be psychometrically sound and to have good validity (Pawlikowski et al., 2013; Tran et al., 2017b). The Internet Addiction Test was originally developed to assess the diagnostic criteria for pathological gambling and different aspects of an associated addiction syndrome according to DSM-IV (e.g. “*Do you feel that you access the Internet more often than you expect?*”; “*Do you neglect household chores to spend more time online?*”; “*Do you snap, yell, or act annoyed if someone bothers you while you are online?*”; “*Do you feel preoccupied with the Internet when offline, or fantasize about being online?*”). Responses to the 12 items (rated on a 5-point Likert scale from “*never*” to “*very often*”) are summed to form a scale assessing the overall severity of pathological Internet use. Although there are no established clinical cut-offs for syndromic diagnosis, Pawlikowski et al. (2013) have made some suggestions regarding content-based and statistical considerations for establishing preliminary/exploratory cut-off values: These authors have suggested that a scale score higher than 37 indicate pathological forms of Internet use in terms of an addiction syndrome.

#### Loneliness Scale (LSC)

The 11-item loneliness scale, developed by Jong-Gierveld and Kamphuls (1985), was used for the assessment of subjective feelings of loneliness. In this model, the experience of loneliness is thought to result from a discrepancy between opted-for and actually established social bonds (de Jong-Gierveld & Kamphuls, 1985). Items of the LSC were scored on a 5-point Likert scale (“*NO!*”, “*no*”, “*more or less*”, “*yes*”, “*YES!*”) and summed to form a single scale score of perceived loneliness.

#### Other scale instruments

Since the presented study was part of a larger survey study, several other scale instruments were used: A German-language version of the 8-item form of the impulsivity scale by Whiteside and Lynam (2001) was used (Kovaleva et al., 2012), a scale to assess insecurities and worries concerning university career-related issues by Seifert (1992), a self-report version of the Alcohol, Smoking and Substance Involvement Screening Test (WHO ASSIST Working Group, 2002), the Generalized Anxiety Disorder Scale (Spitzer et al., 2006), the Rosenberg Self-Esteem Scale (Rosenberg, 1965), a short form of Schwarzer and Jerusalem’s (1999) Global Self-Efficacy scale (Romppel et al., 2013), a German version of the Multidimensional Scale of Perceived Social Support (Zimet et al., 1988), the Perceived Stress Scale (Cohen et al., 1983), the short form of Carver’s Coping Inventory (Carver, 1997), the depression scale of the Patient Health Questionnaire (Kroenke et al., 2001), the MINI Social Phobia Inventory (Connor et al., 2001), and the adult Attention Deficit/Hyperactivity Disorder Self-Report Scale (Kessler et al., 2005).

### Procedure

On accessing the web link, subjects were forwarded to a welcome screen containing information about the content and duration of the survey, a notice of confidentiality and a declaration of informed consent. Upon proceeding to the next screen, the online survey started and participants filled in the described survey instruments. On completion of the survey, participants had the possibility to give their consent and sign up to participate in a raffle offering five prizes of 25 Euros.

### Statistical analyses

**Fig. 2:**
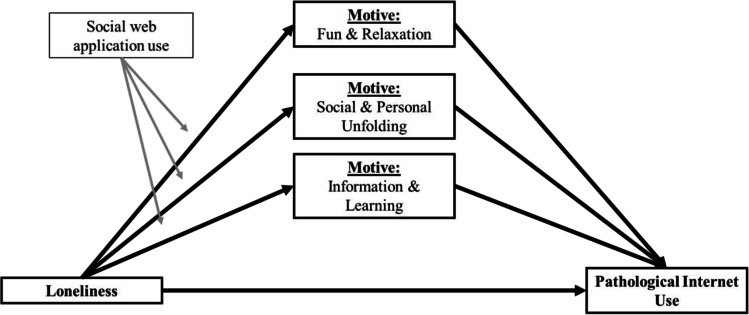
Conceptual model specifying conditional indirect effects of loneliness upon pathological Internet use, as mediated by different types of Internet use motives and moderated by the amount of social web application use

All statistical analyses regarding the hypotheses of the present study were performed using the Statistical Package for the Social Sciences (SPSS, Version 23) with the PROCESS macro (Hayes, 2013, version 2.15) installed. All inferential tests were performed using the standard alpha criterion (i.e. α ≤ .05). Descriptives of all scale scores were calculated assuming metric scale level (i.e. means, standard errors, Cronbach’s alpha, bivariate correlations). In order to investigate the posited research questions, a first stage moderated parallel multiple mediator model was developed for the sIAT score as a criterion measure of pathological Internet use and the three Internet motive dimensions (fun & relaxation, information & learning, social & personal unfolding) as mediating variables (PROCESS Model 7, see the conceptual model in Fig. 2), with social web application use as a moderator of loneliness effects in each of the indirect effect paths (controlling for overall Internet use duration). Identified conditional indirect and total effects were further analyzed and visualized using the PROCESS macro for SPSS (Hayes, 2013). In order to judge the presence of effect contingency (i.e. moderation) in the indirect effect paths, Hayes’ index of moderated mediation (Hayes, 2013, 2015) was used. Statistical significance of conditional indirect (and total) effects were assessed against the background of 95% bias-corrected bootstrap confidence intervals, based on n=10.000 bootstrap samples. All presented regression coefficients will be expressed both in their unstandardized and standardized form.

## Results

### Descriptive statistics

The descriptive statistics for the estimated durations (in hours per week) of service-specific and overall Internet use are presented in Table 2. As can be seen, the whole sample reported a mean duration of almost 35 hours of overall Internet use per week. Approximately 60% of this time (~21h) was devoted to either social web application or audiovisual entertainment use, while other uses made up only minor parts of total online time.

Descriptive statistics concerning employed psychometric scales can be found in Table 3. The mean value for the sIAT scores as indicator of pathological Internet use was 23.84 (SD=6.22), with 11 persons (2.5%) scoring above the diagnostic threshold of 37 for syndromic diagnosis (see Pawlikowski et al., 2013).

**Table 3:** Descriptive statistics of the employed psychometric scales

Instrument	M	SD	Min	Max	Cronbach’s Alpha	Scale Range
M1_fun	11.41	2.52	3	15	.794	3–15
M2_soc	6.79	2.72	3	15	.694	3–15
M3_inf	12.19	1.96	5	15	.654	3–15
sIAT	23.84	6.22	12	44	.816	12–60
LSC	24.14	8.54	11	55	.908	11–55

*M: mean; SD: standard deviation; MIN: minimal participant scale score; MAX: maximal participant scale score.*

**Table 4:** Overview of the 16 primary items of the motive scale, their descriptive statistics, the final three-factorial solution with the allocated items and their factor loadings (based on EFA subsample with n = 219)

Item #	Item wording	Factor Loadings			h^2^	% of variance explained	M	SD
		Factor 1	Factor 2	Factor 3				
	**Factor 1: Fun & Relaxation**					Factor 1		
5^a^	… because it is fun to me	0.83	0.03	0.00	0.71	21.3%	3.98	0.96
4^a^	… because I can relax while online	0.82	-0.07	0.06	0.69		3.67	1.12
12^b^	… because it offers excitement and entertainment	0.65	0.18	0.04	0.54		3.74	0.97
	**Factor 2: Social & Personal Unfolding**					Factor 2		
6^a^	… because then I do not feel lonely	0.10	-0.11	0.76	0.64	18.9%	2.21	1.11
15^b^	… because this is where I can be the real me	0.08	-0.03	0.72	0.56		2.12	1.16
11^b^	… because it is the place, where I get the support I need	-0.03	0.17	0.67	0.47		2.35	1.14
	**Factor 3: Information & Learning**					Factor 3		
3^a^	… because I want to inform myself	0.14	0.81	-0.17	0.74	19.2%	4.45	0.78
2^a^	… because it gives me food for thought	-0.09	0.72	0.31	0.59		3.68	0.99
9^a^	… because thereby I learn about things useful for everyday life	0.03	0.63	-0.08	0.41		4.00	0.85
	excluded items	(values in primary solution)				Overall		
10^b^	… because thereby I can relieve stress	0.65	-0.11	0.29	0.63	59.4%	2.78	1.24
13^b^	… because thereby I can forget about troubles of everyday life	0.58	-0.17	0.37	0.62		2.59	1.22
7^a^	… because I want to distract myself	0.54	0.03	0.14	0.38		3.42	1.13
8^a^	… because it is by habit that it is a part of my life	0.27	0.11	0.23	0.19		3.59	1.16
14^b^	… because it is useful and convenient	0.41	0.30	-0.10	0.30		4.23	0.83
1^a^	… so I can keep up and talk with others	-0.03	0.32	0.31	0.18		2.84	1.13
16^b^	… because thereby I can feel close to persons important to me	0.08	0.02	0.43	0.22		2.80	1.29

^a^: item developed by Engel and Best (2010); ^b^: self-developed item; M: mean; SD: standard deviation.

**Table 5:** Intercorrelations between loneliness and Internet (ab)use related measures

	1	2	3	4	5	6	7
1. LSC	**1**	.112^*^	.087	.328^***^	.338^***^	-.064	.055
2. M1_fun	.112^*^	**1**	.343^***^	.260^***^	.286^***^	.063	.263^***^
3. M2_soc	.328^***^	.343^***^	**1**	.124^**^	.395^***^	.154^**^	.193^***^
4. M3_inf	.087	.260^***^	.124^**^	**1**	.060	-.012	.086
5. sIAT	.338^***^	.286^***^	.395^***^	.060	**1**	.121^*^	.234^***^
6. DUR_CON	-.064	.063	-.012	.154^**^	.121^*^	**1**	.704^***^
7. DUR_OVERALL	.055	.263^***^	.086	.193^***^	.234^***^	.704^***^	**1**

^***^significant at p < .001 (two-tailed); ^**^ *significant at p < .01 (two-tailed);* ^*^ *significant at p < .05 (two-tailed).*

**Table 6:** Unstandardized (b) and standardized (β) regression coefficients, standard errors and model summary information for the constituent parts of the loneliness first stage moderated parallel multiple mediator model

		Criterion																		
		**M1 (M1_fun)**					**M2 (M2_soc)**					**M3 (M3_inf)**					**Y (sIAT)**			
		b	β	S.E.	p		b	β	S.E.	p		b	β	S.E.	p		b	β	S.E.	p
X (LSC)	a_11_	.021	.075	.017	.232	a_12_	.077	.337	.018	< .001	a_13_	.007	.072	.014	.623	c’	.172	.236	.032	< .001
W (DUR_CON)	a_21_	-.050	-.224	.015	.004	a_22_	-.004	.164	.016	.819	a_23_	-.031	-.102	.012	.031		**―**	**―**	**―**	**―**
XW (Interaction)	a_31_	.000	.006	.001	.898	a_32_	.003	.118	.001	.013	a_33_	.001	.053	.001	.300		**―**	**―**	**―**	**―**
M1 (M1_fun)		**―**	**―**	**―**	**―**		**―**	**―**	**―**	**―**		**―**	**―**	**―**	**―**	b_1_	.373	.151	.114	.001
M2 (M2_soc)		**―**	**―**	**―**	**―**		**―**	**―**	**―**	**―**		**―**	**―**	**―**	**―**	b_2_	.558	.244	.107	< .001
M3 (M3_inf)		**―**	**―**	**―**	**―**		**―**	**―**	**―**	**―**		**―**	**―**	**―**	**―**	b_3_	-.133	-.042	.136	.329
C (DUR_OVERALL)	a_41_	.036	.418	.006	< .001	a_42_	.007	.073	.006	.254	a_43_	.011	.160	.005	.020	b_4_	.030	.137	.009	.002
Constant	i_M1_	10.33	**―**	.280	< .001	i_M2_	5.20	**―**	.293	< .001	i_M3_	11.89	**―**	.227	< .001	i_Y_	14.13	**―**	1.812	< .001
		R^2^ = .104 (ΔINT = .000)					R^2^ = .156 (ΔINT = .012)					R^2^ = .025 (ΔINT = .002)					R^2^ = .249			
		F(4, 440) = 12.795, p = < .001					F(4, 440) = 20.306, p = < .001					F(4, 440) = 2.793, p = .026					F(5, 439) = 29.127, p = < .001			

*To facilitate the interpretation of coefficients, a score of 11 was subtracted from each individual’s LSC score before entering it into the models. Hence, a score of 0 in the LSC corresponds to the lowest achievable scale score, indicating the absence of loneliness feelings; ΔINT: change in R*^*2*^ *attributable to the inclusion of the interaction term XW.*

**Table 7:** Decomposed quantification and statistical inference regarding loneliness effects on pathological Internet use (based on unstandardized regression coefficients)

Effect Part		Notation	b	β	BC_LLCI	BC_ULCI
unconditional direct effect part		c'	.1723	.2364	.1011	.2414
conditional indirect effect part	unconditional mediation (a_1i_b_i_)	∑(a_1i_b_i_)	.0497	.0906	.0199	.0906
		M1: (a_11_b_1_)	.0077	.0113	-.0041	.0253
		M2: (a_12_b_2_)	.0430	.0823	.0204	.0791
		M3: (a_13_b_3_)	-.0009	-.0030	-.0129	.0022
	conditional/ moderated mediation (a_3i_b_i_)	∑(a_3i_b_i_)*W	.0017	.0276	-.0005	.0039
		M1: (a_31_b_1_)*W	.0001	.0009	-.0011	.0011
		M2: (a_32_b_2_)*W	.0018	.0289	.0004	.0036
		M3: (a_33_b_3_)*W	-.0001	-.0022	-.0010	.0001

*BC_LLCI: lower limit of the bias-corrected 95% confidence interval of bootstrap sample estimates (based on unstandardized regression coefficients); BC_ULCI: upper limit of the bias-corrected 95% confidence interval of bootstrap sample estimates (based on unstandardized regression coefficients).*

A full display of intercorrelations between variables fed into the statistical model can be found in Table 5. As can be seen, loneliness scale scores were significantly and positively correlated in the weak-to-moderate range with pathological Internet use scale scores and with two of the Internet use motives subscales, namely “*fun & relaxation*”, and “*social & personal unfolding*”. Among the Internet (ab)use indicators, there were weak-to-moderate significant associations between the sIAT scale, the ”*fun & relaxation*” subscale, the “*social & personal unfolding*” subscale, duration of social web application use and overall Internet use Table 6.

### First stage moderated parallel multiple mediator model

**Fig. 3:**
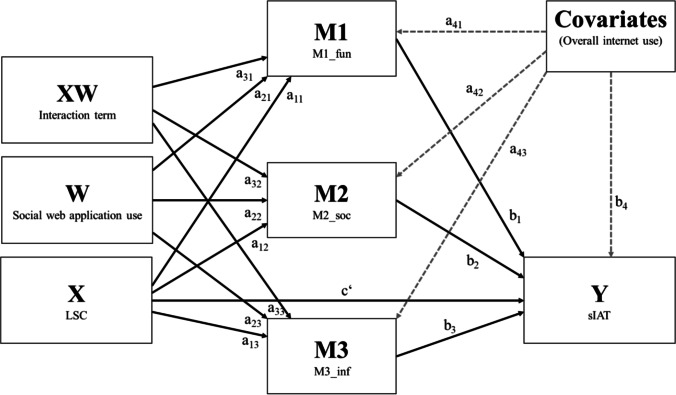
Statistical model specifying the conducted first stage moderated mediation analysis of loneliness effects on pathological Internet use levels, as mediated by different types of Internet use motives and moderated by the amount of social web application use

Figure 3 shows the statistical model of the conducted first stage moderated mediation analysis, the results of which are displayed in Table 7 for both the Internet use motive scales (mediator models) and the pathological Internet use scale (main model).

In the mediator models for each of the three Internet use motive subscales, the included predictors explained between 2.5% and 15.6% of the variance (see Table 7, Models M1 to M3). Only for the model for the “*social & personal unfolding*” subscale, loneliness scale scores were significantly associated with the Internet use motive score (Table 7, Model M2). Moreover, only in this model, there was a significant interaction between loneliness scale scores and social web application use duration (β_a32_=.118; p = .013). This interaction term explained an additional 1.2% of the variance in criterion scores. Probing of this effect showed that loneliness scale scores were more strongly associated with the “*social & personal unfolding*” subscale scores with increasing levels of social web application use. As can also be seen in Table 7, overall Internet use duration was significantly and positively associated with “*fun & relaxation*” and “*information & learning*” subscale scores (Table 7, Models M1 & M3), but not for “*social & personal unfolding*” subscale scores (see Table 7, Model M3).

In the main model of pathological Internet use, both “*fun & relaxation*” and “*social & personal unfolding*” subscale scores were significantly associated with pathological Internet use scores (Table 7, Model Y). Similarly, the direct effect of loneliness was significant and positive (β_c’_=.236; p<.001), as was the effect of overall Internet use.

**Fig. 4:**
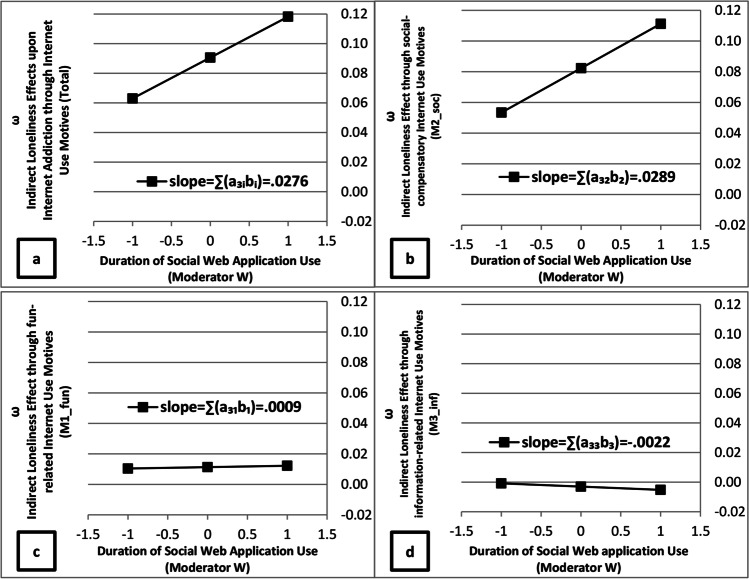
Probing of conditional indirect loneliness effects on pathological Internet use as a function of social web application use (Z-standardized values: mean ± 1 SD) for all mediator paths combined (Panel a) and for each of the mediator paths in isolation (Panels b, c and d), expressed in terms of changes in the size of the standardized regression coefficients for the conditional indirect effect (i.e. a_1i_b_i_ + a_3i_b_i_*W)

In terms of the moderated mediation analyses, conditional indirect loneliness effects were statistically non-significant for both the “*fun & relaxation*” and “*information & learning*” mediator pathways (see Table 7, M1 and M3). In contrast to these findings, the indicators for both the unconditional (β_a12b2_ = .0823) and the conditional parts (β_a32b2_ =.0289) of indirect loneliness effects through the “*social & personal unfolding*” mediator pathway were positive and statistically significant based on 95%-bootstrap confidence intervals (see Table 7, M2). Figure 4 presents a graphical representation of conditional indirect loneliness in terms of changes in the (standardized) regression weights as a function of social web application use duration for all indirect effect pathways combined (Panel a) and for the single indirect effect paths (Panels b to d). As can be seen, conditional indirect loneliness effects occurred specifically in the “*social & personal unfolding*” mediator pathway (Panel d).

## Discussion

The main aim of this study was to put to the test a conceptually extended version of the cognitive-behavioral model of pathological Internet use. Based on the empirical evidence, the sole primacy of social-compensatory Internet use motives in predicting pathological Internet use levels was questioned, and it was hypothesized that other use motives may also be related to the syndrome of pathological Internet use in significant and meaningful ways. This could be confirmed during the moderated mediation analysis conducted, as indicators of “*fun & relaxation*” as well as “*social & personal unfolding*” motives were associated with pathological Internet use. Contrary to our expectations, loneliness had specific indirect effects through social-compensatory Internet use motives. Our hypotheses pertaining to the moderation of indirect loneliness effects could be confirmed, since social web application use moderated loneliness effects on social-compensatory Internet use motives (i.e. “*social & personal unfolding*” subscale scores).

### Loneliness, Internet gratifications and pathological Internet use

#### Specificity of loneliness effects on social-compensatory use orientation

Based on previous findings, we expected to find robust associations between loneliness and Internet use orientations other than social-compensatory ones. However, this could only be shown at the bivariate level for the “*fun & relaxation”* motive subscale, which is generally in line with previous studies (Brand et al., 2014; Caplan, 2002, 2003; Morahan-Martin & Schumacher, 2003). In contrast, loneliness was unrelated to the “*information & learning”* motive subscale, which is consistent with findings from other studies (Matsuba, 2006; Seepersad, 2004, but see Morahan-Martin & Schumacher, 2003). Social-compensatory Internet use orientation, as assessed by a subscale containing items concerning *“social & personal unfolding”*, was positively and moderately associated with loneliness levels both in the bivariate and in the regression analyses presented (see Tables 3 & 5). This replicates findings from earlier studies (Brand et al., 2014; Caplan, 2002, 2003; Hollenbaugh & Ferris, 2014; Morahan-Martin & Schumacher, 2003).

Based on our results, loneliness would indeed seem to be specifically associated with a social-compensatory use orientation, much in the same way as postulated within the cognitive-behavioral model of pathological Internet use (Davis, 2001). Since the present study captured only three different dimensions of Internet use motives, however, these findings should not be taken as definitive. As other researchers have established more than these three domains of use motives as relevant for Internet use (Sundar & Limperos, 2013), future studies of loneliness might try to capture these different constructs in order to arrive at more definite results regarding the specificity of loneliness effects.

#### Usage-contingent associations between loneliness and social-compensatory use motives

As hypothesized (Hypothesis 1b), this study found consistent evidence for the existence of a specific moderation effect pertaining to social-compensatory Internet use motives. This finding of usage-contingent relations between loneliness and social-compensatory Internet use motives warrants further discussion. A study of Kim et al. (2009) found a somewhat similar conditional loneliness effect on social-compensatory use orientation, which was contingent on an individual’s Internet use orientation. These researchers asked their participants to indicate their favorite type of Internet activity and allocated them to one of three favorite activity groups (downloading/streaming files vs. social networking vs. instant messaging). While generally replicating the predictions from the cognitive behavioral model (i.e. social-compensatory use orientation partly mediates loneliness effects on pathological Internet use), Kim et al. (2009) also conducted a multigroup analysis to compare the structural parts of their models between the three groups. Interestingly, they found evidence for a strong direct effect of loneliness on pathological Internet use levels in the download group, with only little indication of effect mediation through social-compensatory Internet use orientation. In contrast, loneliness effects on pathological Internet use were fully mediated through social-compensatory use orientation in the instant messaging group. The social networking group fell somewhere in between the other groups (i.e. partial mediation effect). Note that this evidence is compatible with the findings of the present study, since higher levels of social Internet service use conditioned stronger relations between loneliness and social-compensatory Internet use motives. To the knowledge of the author, this is the only other study that specifically draws on the usage-contingent relations between psychosocial characteristics and media-related user motives in the field of pathological Internet use research. This evidence should be taken to mean that, depending on the precise nature of Internet use adopted by a person, psychosocial factors such as loneliness might be differentially related to different motivational underpinnings of use.

In regard to clinical significance, the present findings may help shed light on the different syndromic expressions of pathological Internet use. If one could show that certain types of Internet use interact with certain psychological characteristics and directly or indirectly influence the development of pathological Internet use, this may improve the understanding of the functional significance of Internet use for the affected individual. A lonely person using the Internet for mood management purposes and spending a lot of time with entertainment watching or online games may be different from a lonely person using the Internet for the actualization of self and spending a lot of time on social web applications. While both may score high on scales assessing the presence of pathological Internet use symptoms, they may differ considerably in their underlying use expectancies and their adopted coping strategies and may therefore require individually tailored treatment approaches. This requires further investigation addressing some of the conceptual and methodological limitations of the present study design.

#### Loneliness and its specific association with pathological Internet use

The first hypothesis derived from research question 2 posited that use motives other than social-compensatory ones would (*i.*) be associated with pathological Internet use and (*ii.*) act as mediators of loneliness effects (Hypothesis 2a). Second, the size of these indirect loneliness effects via Internet use motive domains was hypothesized to be contingent on social web application use intensity (Hypothesis 2b).

The results of the present study showed that social-compensatory use motives were the only motivational dimension mediating the association between loneliness and pathological Internet use, thereby contradicting our Hypothesis 2a (part *ii.*). This finding confirms the specificity-prediction of indirect loneliness effects of the cognitive-behavioral model of pathological Internet use (Caplan, 2003; Davis, 2001) and is generally in line with previous findings (Caplan, 2003; Celik et al., 2014; Kim et al., 2009). Besides that, however, social-compensatory use orientation was not the only domain of Internet-related cognitions/motives that was significantly related to pathological Internet use. Importantly, “*fun & relaxation*” motives were positively associated with pathological Internet use. Therefore, there would appear to be no primacy for one specific kind of use orientation in explaining pathological Internet use, since motives of entertainment, relaxation, arousal and emotion regulation have all been found to be associated with syndrome severity (Brand et al., 2014; Kim & Haridakis, 2009; Morahan-Martin, 1999). This finding clearly points to the fact that the predictions of the cognitive-behavioral model should be broadened to include other motivational domains of Internet use. By doing so, the precise ways by which a broad array of different demographic, psychosocial and mental health factors are related to pathological Internet use may better be explained. The present results showing a specific association between loneliness and pathological Internet use through social-compensatory Internet use motives (contingent on the level of social web application use adopted) might be taken as one such pathway to the potential development of the disorder. Given the reported associations of the syndrome with different psychopathological conditions (Ho et al., 2014; Kuss et al., 2014; Sun et al., 2012), it may well be associated with Internet use motivations other than social compensatory ones, clearly pointing to the need of additional research regarding the motivational underpinnings underlying the development of pathological Internet use in the affected individual.

### Methodological and conceptual limitations

While interesting in their implications, the present results need to be qualified against the background of some methodological and conceptual drawbacks. First, as the sample was restricted to university students, the generalizability of the present findings needs to be discussed. In our sample, a total of 2.5% of participants scored above the diagnostic threshold for pathological Internet use. These figures are comparable to those found in more representative studies of adolescents in Germany (e.g. Durkee et al., 2012), although they were a little lower. Therefore, the present findings should be replicated in community-based samples. A second issue is the non-consideration of measurement error in the employed statistical analyses, which could be remedied using latent variable modeling techniques such as structural equation modeling (Kline, 2015). A third issue concerns the adopted cross-sectional study design. The present findings should clearly be replicated and qualified by longitudinal studies. Another important issue is the timing of the present study. Since data collection was conducted before the COVID-19 pandemic, the generalizability of the reported findings to the current global situation needs to be considered. Since enforced social isolation due to lockdown measures could have intensified both feelings of loneliness and levels of Internet use, the influence of environmental context needs to be considered when discussing the present results. A recent study of children and adolescents in China has shown an increasing frequency and duration of Internet use (Dong et al., 2020). In this study, levels of pathological Internet use were associated with levels of stress and depression (Dong et al., 2020). Furthermore, a recent meta-analysis has shown that lockdown measures led to significantly increased depression levels, while effects were non-significant for loneliness (Prati & Mancini, 2021). Therefore, it is currently unclear whether increased levels of Internet use resulting from enforced social isolation may have been driven by increased levels of loneliness. Nevertheless, social web application use as a consequence of (enforced) social isolation may have an important and lasting impact on Internet use motives including social-compensatory Internet use motives. Whether or not these motives are related to pathological Internet use levels under conditions of externally enforced social isolation in the same way as in the present study sample is an important question that should be investigated in future research studies. Answering these questions may help shed some light on another important aspect regarding the study of pathological Internet use as a diagnostic entity, i.e. the role of environmental context in the etiology of pathological forms of Internet use.

### Concluding remarks

The findings of the present study highlight the importance of considering actual Internet use behavior (by type and quantity) in understanding the associations between psychosocial factors such as loneliness and pathological Internet use. Moreover, the findings of the present study question the narrow focus on social-compensatory Internet use motives and call for a broader recognition of different Internet use motive dimensions in etiological models of pathological Internet use, such as the cognitive-behavioral model. A usage-sensitive perspective may translate into clinical practice, with a specific analysis of affected individuals’ Internet use behaviors (as well as underlying use motives) being an avenue for targeted therapeutic interventions.

## Data Availability

The datasets generated during and/or analyzed during the current study are available from https://osf.io/8jfec/.
